# Supplementary material for: Mediating effects of general self-efficacy on the relationship between the source of meaning in life and prosocial behaviours in vocational college nursing students: A cross-sectional study
Source: PLoS One. 2020 Dec 15;15(12):e0243796. doi: 10.1371/journal.pone.0243796 (PMC7737967; doi:10.1371/journal.pone.0243796)
Supplement: Attachment — Submitted filename: revison.docx [file pone.0243796.s002.docx]

Thank you for the possibility to review this article. This study is very interesting for nursing educators and the quality of care for patients. However, I have some comments:

1) It could be interesting to have more critical background about general self-efficacy, the source of meaning in life (SML) and prosocial behaviours. Are there previous studies examining these dimensions? In a population of students? Or in Nursing Students? Which were the finding? There are similar models in other studies?
2) Why there is the need to improve the humanistic care quality among nursing students? can you give some information about this sentence? It is not clear in which way the general self-efficacy, the source of meaning in life (SML) and prosocial behaviours are linked with improvement of humanistic care quality among students.
3) Why in introduction about students’ characteristics? I have not clear the reasons for these information.
4) You had hypothesized a relation between general self-efficacy, the source of meaning in life (SML) and prosocial behaviours and for these reasons you had tested the model. But on the basis of what? Are there previous studies?
5) You said “*Studies show that adolescents with higher prosocial behaviour are more responsible and sympathetic than those without such behaviour, are good at perspective taking, and have relatively high levels of prosocial moral reasoning*” but in your study you are speaking about nursing students and not adolescents. Are there other studies about student’s population? about health care workers?
6) In pag 1, line 31 there are two and.
7) About the instruments there are only few information. GSES come from WANG but in which kind of population was it validated? In students? Nursing students? If not, there is the necessity to validate the GSES in nursing students. Can we have some information about explorative factor analysis with the identification of the dimensions and about confirmative factor analysis? You used the total score of the GSES but there is a secondary order factor? At the same way it should be reported information about SMLS and PBS.
8) In table 1 there are the differences in prosocial behaviour and demographic characteristics. Why we have not the same information about general self-efficacy and SLM?
9) The paper need more critical discussion. Which is the implication of these finding? Can you work on general self-efficacy to improve the humanistic care quality among nursing students? in which way?
10) In pag 15, line 270 there is ERROR??

Thank you very much. I think this study is very interesting and can be improved.
